# 48-Week effectiveness and tolerability of dolutegravir (DTG) + lamivudine (3TC) in antiretroviral-naïve adults living with HIV: A multicenter real-life cohort

**DOI:** 10.1371/journal.pone.0277606

**Published:** 2022-11-21

**Authors:** Alfonso Cabello-Ubeda, Juan Carlos López Bernardo de Quirós, Luz Martín Carbonero, Jesús Sanz, Jorge Vergas, Álvaro Mena, Miguel Torralba, Marta Hernández Segurado, Adriana Pinto, Francisco Tejerina, Esmeralda Palmier, Ángela Gutiérrez, Pilar Vázquez, Federico Pulido, Miguel Górgolas

**Affiliations:** 1 Division of Infectious Diseases, Fundación Jiménez Díaz University Hospital, Research Health Institute, Universidad Autónoma de Madrid (IIS-FJD, UAM), Madrid, Spain; 2 Gregorio Marañón University Hospital, Infectious Diseases, Madrid, Spain; 3 CIBERINFEC, ISCIII-CIBER of Infectious Diseases, Instituto de Salud Carlos III, Madrid, Spain; 4 La Paz University Hospital, Infectious Diseases, Madrid, Spain; 5 La Princesa University Hospital, Infectious Diseases, Madrid, Spain; 6 Clínico San Carlos University Hospital, Infectious Diseases, Madrid, Spain; 7 A Coruña University Hospital, Infectious Diseases, A Coruña, Spain; 8 Guadalajara University Hospital, Infectious Diseases, UAH, Guadalajara, Spain; 9 12 Octubre University Hospital, imas12, UCM, CIBERINFEC, HIV Unit, Madrid, Spain; Imperial College London, UNITED KINGDOM

## Abstract

**Background:**

The main international guidelines indicate DTG/3TC therapy as one of the preferred regimens for people living with HIV (PLWH), due to its observed efficacy in randomized clinical trials. However, information in real-life cohorts is relatively scarce for first-line use.

**Methods:**

A retrospective multicenter study of adult PLWH starting DTG+3TC as a first-line regimen before January 31^st^, 2020. Virological failure (VF) was defined as 2 consecutive HIV RNA viral load (VL) >50 copies/mL.

**Results:**

135 participants were included. Treatment was started without knowing baseline drug resistance testing (bDRT) results in 71.9% of cases, with baseline resistance mutations being later confirmed in 17 patients (12.6%), two of them with presence of M184V mutation. Effectiveness at week 48 was 85.2% (CI95%: 78.1–90.7%) (ITT missing = failure [M = F]) and 96.6% (CI 95%: 91.6–99.1%) (per-protocol analysis). Six patients (4.4%) discontinued treatment. One developed not confirmed VF after discontinuing treatment due to poor adherence; no resistance-associated mutations emerged. Three discontinued treatments due to central nervous system side effects (2.2%), and two due to a medical decision after determining the M184V mutation in bDRT. Finally, 14 (10.4%) were lost to follow-up, most of them due to the COVID-19 pandemic.

**Conclusions:**

In a real-life multicenter cohort of ART-naïve PLWH, treatment initiation with DTG + 3TC showed high effectiveness and favorable safety results, comparable to those of randomized clinical trials, without treatment-emergent resistance being observed through week 48. Starting treatment before receiving the results of baseline drug resistance testing did not have an impact on the regimen’s effectiveness.

## Introduction

The main international HIV/AIDS treatment guidelines recommend offering antiretroviral treatment (ART) to all people living with HIV (PLWH) as soon as possible. Although classic ART initiation regimens generally contain two nucleoside reverse transcriptase inhibitors (NRTIs) as core therapy, plus a third major agent [1–3], in recent years a two-drug regimen (i.e. dolutegravir [DTG] plus lamivudine [3TC]) has been recommended as a first-line regimen for the treatment of ART-naive patients. This recommendation is based on the results of the GEMINI-1 and -2 phase III clinical trials, which demonstrated long-term non-inferior efficacy of DTG/3TC vs DTG + tenofovir disoproxil fumarate (TDF)/emtricitabine (FTC) in treatment-naive participants [4–6].

Although the emergence and development of combination antiretroviral treatment has improved life expectancy for PLWH, with HIV itself becoming a chronic infection, it has also given rise to an increased incidence of HIV-associated comorbidities, including serious non-AIDS events (SNAEs) [7–9]. As these comorbidities are sometimes related to effects of ART [10, 11], reducing the amount of antiretrovirals taken could potentially be a way to improve the quality of life for PLWH, the ‘fourth 90’ proposed by several authors [12–14].

In this context, DTG/3TC therapy is currently recommended as one of the preferred treatments for PLWH. However, information in real-life cohorts is still scarce. Our aim is to evaluate the effectiveness and tolerability of DTG/3TC in treatment-naïve adult PLWH from a a multicenter real-life cohort.

## Materials and methods

We carried out an observational, two-phase, multicenter study across eight centers in Spain. The first phase was a retrospective cohort with 48-week follow-up from initiation of treatment (second phase will continue a prospective follow-up to 96 weeks). All ART-naïve adult PLWH who initiated DTG+3TC as first line ART regimen in the participant centers before January 31^st^, 2020, were included. Patients taking DTG and 3TC as separate pills or DTG/3TC as a single-tablet regimen were included.

Baseline demographic data, such as gender, country of origin, age, risk factors for HIV infection, and baseline HIV-related data such as CD4+ cell count, CD4/CD8 ratio, HIV-1 RNA viral load, genotypic drug resistance testing and other biochemical parameters including lipid profile and hepatic and renal function were collected. Follow-up visits after therapy initiation included appointments at 4, 12, 24 and 48 weeks (a window period of 4 weeks was allowed for each visit due to the observational nature of the study and non-standardized appointment times).

Adverse events (AEs) registered at the follow-up visits were recorded. The primary endpoint was the proportion of participants with viral suppression (HIV RNA <50 copies/mL) at week 48, using an intention to treat (ITT) analysis considering missed cases (treatment discontinuation for any reason, loss to follow-up, withdrawn informed consent or death from any cause) as failures (M = F). An additional per-protocol analysis was performed excluding those participants who were lost to follow-up and those changing therapy due to the presence of M184V mutation. Virological failure (VF) was defined as two consecutive HIV RNA >50 copies/mL. As Spanish national guidelines allows starting ART without having the results of baseline HIV genotype testing, we recorded the proportion of patients who started ART without this result according to routine clinical practice at each hospital [15].

Ethics committee approval was obtained from an Institutional Review Board (Approval no. EO 119–20), and the study was registered at ClinicalTrials.gov (NCT04638686). Data for the study were collected retrospectively from patients’ medical records, anonymized, and entered into an on-line electronic database. All research was carried out in accordance with the right to privacy, as stipulated in the Organic Regulation (EU) 2016/679 of the European Parliament and of the Council of April 27, 2016 on data protection (GDPR), on the protection of personal data, and the Declaration of Helsinki. A waiver of consent (for retrospective follow-up) from the Ethics Committee was granted as only de-identified data were extracted from clinical records.

Categorical variables were presented as frequencies and were compared using Fisher’s exact test or Pearson’s chi-squared test as appropriate; continuous variables were presented as mean and standard deviation or median and interquartile range (IQR) and were compared using the Student’s t test or the Mann-Whitney U test. Comparisons between baseline and follow-up variables were performed with the Wilcoxon signed-rank test. All statistical analyses were performed using IBM SPSS version 20 and a significance level of .05.

This data has been previously partial communicated in XII Spanish HIV Conference in 2021 [16].

## Results

One hundred and thirty-five participants were included. Baseline characteristics are described in Table 1. Eleven (8.1%) were 50 years-old or older, and 9.6% were women. Twenty-three (17%) presented more than 100,000 copies/mL (one with > 500,000 copies/mL) and 26% had a CD4+ cell count of less than 350 cells/mm^3^ (3 with < 200 cells/mm^3^). Thirty-eight (28%) of them had occasionally taken recreational drugs, while 14% reported practicing *chemsex*.

**Table 1:** Patients’ baseline characteristics.

Baseline characteristics		Patients (N = 135)
Age (years) (median–IQR)		32 (27–39)
Gender, n (%)	Male	122 (90.4)
	Female	13 (9.6)
HIV transmission, n (%)	MSM	112 (83)
	MSW	20 (14.8)
	IDU	3 (2.2)
Country–region, n (%)	Spain	69 (51.1)
	Latinamerican	53 (39.3)
	Europe	6 (4.4)
	Others	7 (5.2)
CDC classification, n (%)	A	125 (92.6)
	B	7 (5.2)
	C	3 (2.2)
CD4+ cells/mm^3^ (median–IQR)	Basal CD4+	469 (347–650)
	CD4 < 200	3 (2.2)
HIV-1 VL (c/mL), n (%)	≥ 100,000 copies/mL	23 (17)
	< 100,000 copies/mL	112 (83)
Hepatitis B co-infection, n (%)	HBsAg +	0
	Anti-HBc +	32 (23.7)
	Anti-HBs +	86 (63.7)
Hepatitis C co-infection (IgG HCV +), n (%)		7 (5.2)
HLAB*5701 positive, n (%)		11 (8.1)
Median time from diagnosis to start of treatment (weeks)		6 (IQR: 3–14)

IQR = interquartile range; MSM = Men who have sex with men; MSW = Men who have sex with women; IDU = injecting drug users; VL = viral load; HBsAg = Hepatitis B surface antigen; Anti-HBc: Antibodies to the Hepatitis B core antigen.

Treatment was started before receiving the results of baseline drug resistance testing (bDRT) in 71.9% of cases, with baseline resistance mutations posteriorly confirmed in 17 (12.6%), two of them with a M184V mutation. In one other case, an integrase inhibitor resistance-associated mutation (RAM) (G163K) was present, although this mutation does not appear to reduce susceptibility to DTG. A total of five bDRTs showed nucleoside RAMs (41L, 44D, 215D/C/S, 210W), 12 non-nucleoside RAMs (138A/G, 103N, 106I, 190A) and one, a protease inhibitor RAM (82C) (Table 2). Treatment was initiated prior to receiving results of CD4+ cell counts, HIV-1 viral load, HBsAg or HLA B*5701 results in 10.4%, 17%, 6.7% and 64.9% of participants, respectively.

**Table 2:** Mutations detected in the baseline drug resistance testing (bDRT); global results and results by patient.

**Baseline characteristics**			**Patients (N = 135)**	
Major mutations in Baseline drug resistance testing (bDRT), n (%)	17 (12.6)	INSTIs	1 (0.7)	G163K
		PIs	1 (0.7)	V82C
		NNRTIs	12 (8.9)	E138A(3), K103N(6), V106I(3), V108Vi, E138EG, G190A
		NRTIs	5 (3.7)	**M184V** (2), M14L, E44D, T215D, T215C, L210W, T215S
**Results by patient**				
**Participant**	**Major mutations in bDRT**		**Antiretroviral family affected**	
1	G163K		INSTIs	
2	E138EG		NNRTIs	
3	M14L, E44D, T215D		NRTIs	
4	K103N		NNRTIs	
5	K103N		NNRTIs	
6	L210W, T215S		NRTIs	
7	E138A		NNRTIs	
8	V106I		NNRTIs	
9	V106I, G190A		NNRTIs	
10	K103N, E138A		NNRTIs	
11	V106I		NNRTIs	
12	K103N, M184V		NNRTIs and NRTIs	
13	M184V		NRTIs	
14	V82C		PIs	
15	K103N, V108Vi, T215C		NNRTIs and NRTIs	
16	E138A		NNRTIs	
17	K103N		NNRTIs	

bDRT: Baseline drug resistance testing; INSTIs = integrase strand transfer inhibitors; PIs = protease inhibitors; NNRTIs = non-nucleoside reverse transcriptase inhibitors; NRTIs = nucleoside reverse transcriptase inhibitors.

**Fig 1:**
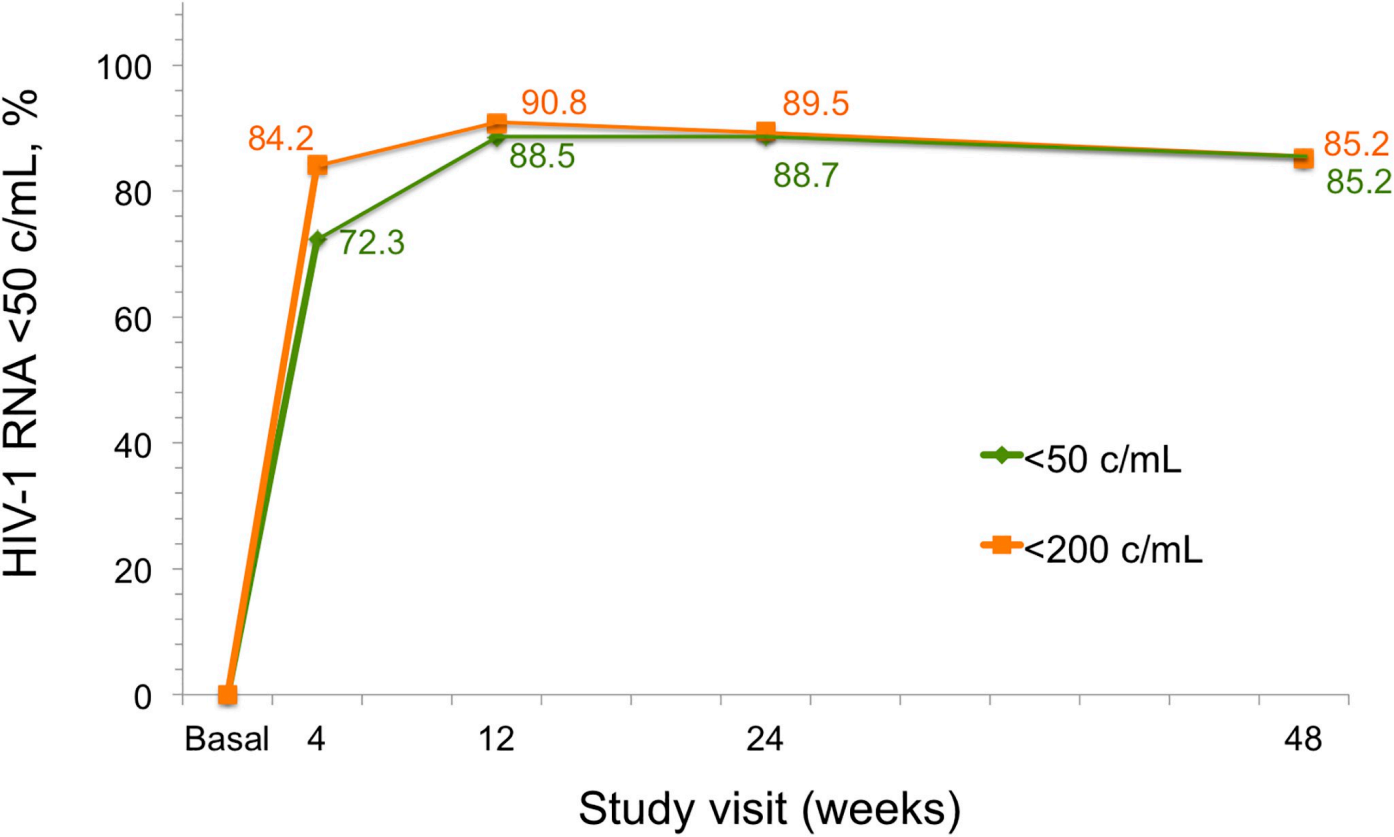
Proportion of participants with plasma HIV-1 RNA <50 c/mL through week 48 by intention to treat (missing = failure) analysis.

Effectiveness at week 48 was 85.2% (115/135; CI95%:78.1–90.7%) and 96.6% (115/119; CI95%: 91.6–99.1%) in the ITT (M = F) and in the per-protocol analysis respectively The proportion of participants with plasma HIV-1 RNA <50 copies/ml through week 48 by ITT (M = F) analysis is illustrated in Fig 1. Six patients discontinued treatment. One developed VF (one HIV-1 VL of 409 copies/mL) after discontinuing treatment; no RAMs emerged. This patient is currently virologically suppressed again on DTG/3TC. Three patients discontinued treatment due to central nervous system (CNS) side effects (2.2%); these symptoms (insomnia, anxiety and headache) resolved after changing the regimen in all cases. DTG/3TC was discontinued in two patients after the M184V mutation was detected in bDRT; one of them had already reached VL <50 copies/mL by week 6 (Table 3). Finally, 14 patients (10.4%) were lost to follow-up, 10/14 on a date related to the first COVID-19 pandemic wave. The disposition of patients at week 48 is summarized in Table 4.

**Table 3:** Patients who discontinued treatment with DTG+3TC.

Patient	Time to ART change (weeks)	Reason	Basal VL (c/mL)	w4	w12	w24	Final ART	Last VL (c/mL)
1	-	VF^a^	779	-	-	409	DTG/3TC	<50
2	1	M184V^b^	1,875	<50	-	-	DRV/c/TAF/FTC	<50
3	2	CNS AE	142	-	-	-	RPV+TDF/FTC	<50
4	6	M184V^c^	29,300	<50	-	-	DTG/RPV	<50
5	16	CNS AE	219	-	<50	-	DRV/c/TAF/FTC	LTFU
6	20	CNS AE	1,696	<50	<50	-	RPV/TAF/FTC	<50

ART: Antiretroviral treatment; VL: viral load; CNS AE: Central nervous system adverse event; VF: virological failure; LTFU: lost to follow-up.

^a^VF according to physician’s criteria (only one VL ≥ 200 copies/mL but the patient stopped treatment).

^b^The participant had HIV-1 RNA 1,875 c/mL at baseline, switched to DRV/c/TAF/FTC on Day 8; <50 copies/mL on Day 59.

^c^The participant had HIV-1 RNA 29,300 c/mL at baseline, <50 copies/mL on Day 43, switched to DTG+DRV/c on Day 43; switched to DTG/RPV afterwards.

**Table 4:** Disposition of the participants at week 48.

All participants	135
HIV-1 VL < 50 c/mL, n (%)	115 (85.2)
DISCONTINUATION, n (%)	6 (4.4)
• HIV-1 VL > 200 copies/mL	1 (0.7)
• CNS AE	3 (2.2)
• M184V mutation in bDRT	2 (1.5)
LOST TO FOLLOW- UP, n (%)	14 (10.4)

AE = adverse event; CNS = Central nervous system adverse event; VL = viral load; bDRT = Baseline drug resistance testing.

The median time from diagnosis to treatment initiation was six weeks (IQR: 3–14). The median CD4+ and CD4/CD8 T-cell ratio increase was 256 cells/mm3 (IQR 157–463 cells/mm3) and 0.28 (IQR 0.10–0.50) respectively, and the median decrease in the estimated glomerular filtration rate was 11.7 ml/min (IQR 5–24.8 ml/min), with all values remaining above 70 mL/min. There were no significant changes in the lipid profile.

**Fig 2:**
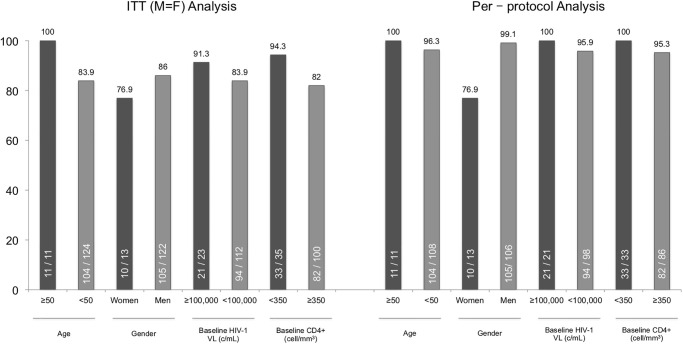
Week 48 pooled intention to treat (missing = failure) and per-protocol analysis stratified by age, gender, baseline HIV-1 viral load and CD4+ cell count.

In the stratified analysis by age, gender, baseline HIV-1 viral load and CD4+ cell count we observed several differences, although without statistical significance (Fig 2). Only three patients presented a baseline CD4+ cell count of less than 200 cell/mm^3^, all of whom continued treatment with DTG/3TC at week 48. All patients with more than 100,000 copies/mL continued treatment with DTG/ 3TC at week 48 except two who were lost to follow-up.

## Discussion

Our findings reflect a high effectiveness and good safety profile for DTG/3TC as initial therapy of PLWH up to week 48 analysis. These results are similar to those observed in randomized clinical trials (GEMINI I and II studies) [6]. Only one patient presented virological failure after discontinuing treatment, without emerging resistance-associated mutations being detected. Only three patients discontinued treatment due to adverse drug reactions.

To the best of our knowledge, the few similar cohorts reported in the literature [17–21] show comparable findings to those of our study. One study does not describe any cases of VF or AE [17], while other reports only one AE and one case of detectable viral load at 24-week analysis [18]. Both cohorts included a significantly lower number of patients than the present study, similarly to other small cohort comparing DTG/3TC with DTG/ABC/3TC [22]. Two systematic literature reviews of patients initiating therapy with DTG/3TC have recently been published and communicated [23, 24], but the number of naïve participants is small (69) [23] and follow-up periods are heterogenous. Nevertheless, this review also reports similar results in terms of efficacy, tolerability, and discontinuation rates due to AE, toxicities, and intolerance with dolutegravir plus lamivudine. A recent meta-analysis evaluating the effectiveness and safety of the regimen in virologically suppressed patients also shows high success rates [25].

It is important to note that 72% of the participants in our cohort started treatment before receiving baseline drug resistance testing results. We can only compare these figures with those from the recently reported STAT study [26, 27], This study suggests that initiating treatment with DTG/3TC prior to receiving bDRT results or data related to HIV-1 viral load or CD4+ cell count does not have a negative impact on patient outcomes, maintaining high levels of effectiveness and safety. Similarly, in our cohort, patients who switched regimens due to presence of the M184V mutation on bDRT maintained undetectable viral load control; one patient even presented an undetectable viral load after 6 weeks on DTG/3TC.

The only case which we have considered virological failure was a patient who stopped treatment of her own accord, with a single viral load > 200 copies/mL observed prior to changing therapy. After receiving other therapies, this patient resumed treatment with DTG/3TC, without further lack of optimal adherence, maintaining good HIV-1 viral load control. This finding is consistent with the high barrier to resistance of dolutegravir plus lamivudine observed in phase III randomized controlled clinical trials, where only one case of virological failure has been reported, with treatment-emergent resistance associated with poor adherence to medication [6].

Adverse events were all mild and very infrequent, as reported in other studies. However, due the retrospective design of the cohort, only those adverse events that cause discontinuation of the therapy have been consistently registered, and other adverse events could be underreported.

Stratified analyses in patients with HIV-1 viral load >100,000 copies/mL (with one patient presenting >500,000 copies/mL) do not show significant differences, maintaining similar or higher effectiveness rates than those with viral loads <100,000 copies/mL. These data is similar to those reported in randomized clinical trials and reflect that treatment with DTG/3TC maintains maximum levels of effectiveness even in this setting. Stratified analyses by gender, age and CD4+ level did not show significant differences, although there were too few patients with CD4+ cell counts <200 cell/mm^3^ to draw conclusions in this subgroup (only three participants presented <200 CD4+ cell/mm^3^, [113, 145 and 189 cell/mm^3^] all of whom continue with DTG/3TC).

Our study has some limitations. First, the retrospective nature of the study inherently leads to a certain risk of loss of information and channeling bias in the characteristics of patients who start this new kind of therapy. However, as all the participants initiating DTG+3TC as first therapy were included, our cohort reflects the actual group of patients who are starting this treatment in a real-life scenario. A second limitation concerns the loss of participants due to the COVID-19 pandemic. Also, the limited number of participants in the stratified analysis does not permit statistically significant conclusions to be drawn in these scenarios. Another limitation is that our study is a single arm cohort and we do not have the context information when comparing it with a control group. Despite this limitation, we have discussed our results comparing with those of most publications, either randomized clinical trials and cohort studies, and our results endorse the data found by other authors.

Despite these limitations, we believe that our work adds some very novel data to the current literature on the use of DTG/3TC therapy in ART-naïve patients. To our knowledge, it is the first multicenter cohort to describe real-life 48-week follow-up data in this setting, in addition to being the largest cohort of treatment-naïve adult patients treated with DTG/3TC outside of randomized clinical trials, and including patients before having baseline drug resistance testing results. We did not observe differences among subgroups (> 50 years-old, HIV-1 viral load >100,000 copies/mL, women, and others). However, the number of them included does not allow us to determine significant differences. Further studies are needed to confirm our results in these underrepresented subgroups, as those with lower CD4+ counts or very high viral loads. It is also necessary to confirm the long-term efficacy and tolerability of the regimen outside of randomized clinical trials.

## Conclusion

In a real-life multicenter cohort of ART-naïve PLWH, treatment initiation with DTG+3TC showed high effectiveness and safety, similar to that of randomized clinical trials, without treatment-emergent resistance at 48 weeks. Starting treatment without the results of the baseline drug resistance test did not have an impact on the regimen’s effectiveness.

## Ethics approval and consent to participate

A waiver of consent from the Ethics Committee was granted as only de-identified data were extracted from the medical records. Approval was obtained from the institutional research ethics committee (Approval no. EO 119–20 from the Fundación Jiménez Díaz Medical University Hospital Ethics Committee, Community of Madrid, Spain). All research was performed in accordance with the General Data Protection Regulation (GDPR) Regulation (EU) 2016/679 of the European Parliament and of the Council of 27 April 2016 on the protection of natural persons with regard to the processing of personal data (General Data Protection Regulation) and the Declaration of Helsinki.

## Supporting information

S1 Graphical abstract Visual abstract. (TIFF)
