# Supplementary figures and images for: 48-Week effectiveness and tolerability of dolutegravir (DTG) + lamivudine (3TC) in antiretroviral-naïve adults living with HIV: A multicenter real-life cohort
Source: PLoS One. 2022 Nov 21;17(11):e0277606. doi: 10.1371/journal.pone.0277606 (PMC9678299; doi:10.1371/journal.pone.0277606)

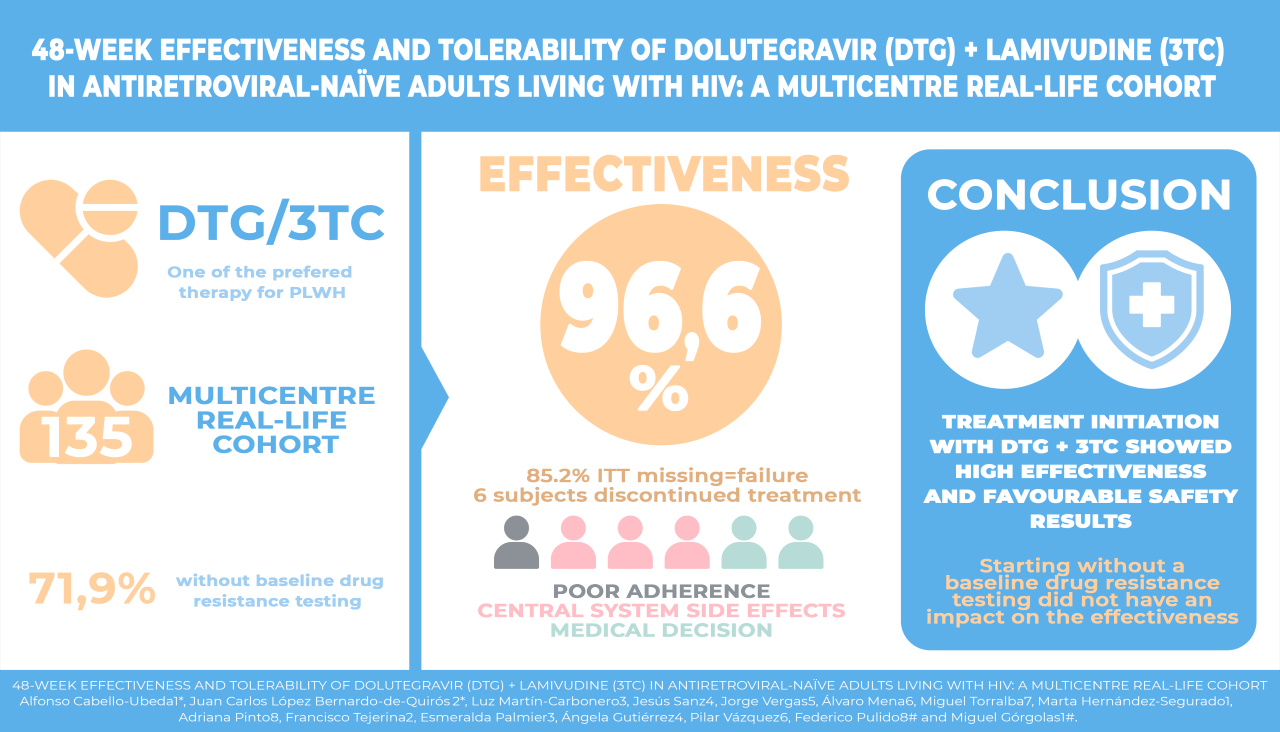

Supplement: S1 Graphical abstract — (TIFF) [file pone.0277606.s001.tiff]
